# Strengthening equitable research capacity in response to infectious diseases of poverty

**DOI:** 10.1136/bmjgh-2026-023520

**Published:** 2026-03-19

**Authors:** Emmanuel Asampong, Maria Isabel Echavarria Mejia, Yodi Mahendradhata, Mahnaz Vahedi, Anna Thorson

**Affiliations:** 1 School of Public Health, University of Ghana, Accra, Greater Accra Region, Ghana; 2 CIDEIM, Cali, Valle del Cauca, Colombia; 3 Gadjah Mada University, Yogyakarta, Jogja, Indonesia; 4 UNICEF/UNDP/World Bank/WHO Special Programme for Research and Training in Tropical Diseases (TDR), Geneva, Switzerland; 5 UNICEF/UNDP/World Bank/WHO Special Programme for Research and Training in Tropical Diseases (TDR), World Health Organization, Geneva, Switzerland

**Keywords:** Health policy

## Abstract

The inequitable global distribution of resources for research parallels the unequal global distribution of morbidity and mortality due to infectious diseases. Significant gaps in research capacity prevail, and equitable and accessible opportunities for research remain a priority. We argue for the democratisation of research: without equitable participation in, and ownership of, research, by those who are implementing the research or are part of the communities being researched, contextualised research needs and health system bottlenecks will remain unresolved. This perpetuates an inequitable power balance related to research and innovation. Equitable research capacity is fundamental to tackling global health challenges and reducing health inequity. We emphasise the evolution from externally driven, high-income-centric models of research capacity strengthening towards inclusive, context-sensitive approaches that prioritise local ownership, diversity and sustainability. A paradigm shift from ‘imposing technical support’ to ‘fostering ownership of knowledge’ has catalysed new models of engagement, such as implementation research capacity among health professionals and communities, and regionally anchored postgraduate training. Institutionalised, inclusive research can align with national priorities and yield measurable improvements in health outcomes. However, persistent inequities rooted in gender, geography and institutional hierarchies continue to constrain participation and impact. Addressing these requires deliberate strategies to democratise access, diversify partnerships and support under-represented institutions and individuals. Allowing dynamic roles in long-term partnerships and regional networks on a continuum between academic partners and capacity-strengthening recipients can support mitigation of intersectional inequities and lead to capacity strengthening.

**Summary box**

- Local ownership and contextualisation should be prioritised in research capacity strengthening.
- Access to research training beyond academia needs to be expanded.
- Research capacity building should be embedded in governance structures and supported with sustainable resources.

## Introduction

 ‘Equitable research capacity’ is the fair distribution of resources, opportunities and power among individual researchers and institutions so that they can fully participate in, and benefit from, research. It is fundamental to tackling global health challenges and reducing health inequities.^1^ Research is necessary to drive evidence-based health improvement, but in many low-income and lower-middle-income countries, its use and application are challenged by a paucity of skilled human resources and limited policy support for institutions. Global funding for science is distributed inequitably, mostly favouring high-income countries (HIC). In low-income and lower-middle-income countries, national research governance, resources and management show systemic gaps, such as insufficient regulatory frameworks, lack of coordinated research policies, limited infrastructure and inadequate administrative support.^1^

To counteract this and to improve health outcomes in low and middle-income countries, multilateral organisations and health donors have in recent decades prioritised ‘research capacity strengthening’ to enhance the abilities of individuals and institutions to conduct and apply health research, including through training, infrastructure development, institutional strengthening and supportive policies.^2^

This *BMJ Global Health* collection highlights the evolution of inclusive internationalism over the past 50 years through the lens of the Special Programme for Research and Training in Tropical Diseases (TDR), co-sponsored by the United Nations Children’s Fund, the United Nations Development Programme, the World Bank and the WHO. Inclusive internationalism is a collaborative approach that ensures fair representation and active participation of all countries in global health initiatives, research partnerships and policy making, emphasising equitable sharing of resources, knowledge and decision-making power to address health inequities and promote collective solutions to global health challenges.^3^ This article aims to identify gaps and opportunities for future strengthening of research capacity to promote equitable participation, relevance and quality of regional, national and global health research.

## Research capacity strengthening then and now

Inequities and power imbalances in global health research have been well described, applying decolonisation perspectives.^4^ Historically, activities to strengthen research capacity were often ‘embedded’, meaning that research projects led by institutions from HIC were implemented together with local counterparts, applying a ‘learning by doing’ approach. Research agenda setting, data analysis and scientific authoring tended to be led by HIC institutions, at the expense of the implementing institutions in lower-income countries.

Global health and development actors have hence argued for a fundamental shift in research agenda setting, from top-down approaches to facilitating ownership of knowledge. Approaches emphasise focus on the agency and capacity of implementing researchers and institutions to ensure active contribution and leadership in asking the research questions, generating and using knowledge instead of risking roles as more passive recipients.^5^

Universal support, including political commitment, sustainable funding and collaboration from national governments, health systems, global funders and research institutions, is important to achieve more equitable research capacity. Expanded and inclusive leadership, participation and access to research outcomes are critical to maximise efficiency and counteract intersectional inequities.

The distinction that research partnerships are either ‘academic collaborations’ or ‘research capacity-strengthening recipients’ is challenged by TDR applying a continuum approach to partnerships. Partners’ roles are not static as recipients or providers, but include dynamic and needs-based features, where TDR regional research training centres^6 7^ and Universities offering the Implementation Research focused programme for MSc in Public Health^8^ aim to strengthen capacity and impact health beyond national borders. Dedicated courses and learning projects targeting health professionals and communities and strengthening advanced implementation research respond to a range of capacity needs within health programmes and academia alike. Selection of beneficiaries of fellowships, graduate programmes, institutional mentoring or supported learning projects is monitored for equitable implementation.

## Expanding equitable academic training

Historically, capacity-strengthening programmes most often have recruited talented students from low-income and middle-income countries (LMIC) for research training in institutions located in HIC in North America, Europe or in Australia. In support of more equitable research capacity-strengthening strategies, TDR in 2015 moved its MSc in Public Health programme, which focuses on implementation research and infectious diseases of poverty, from HIC institutions to instead be run by eight universities in LMIC. These universities were competitively selected, representing implementation research strength and geographical diversity.^8^ Candidates are selected based on merit, gender and geographical affiliation, with the goal to strengthen equitable and diverse research capacity. To date, the TDR MPH training scheme counts 536 grantees, including 50% women from 80 LMIC.^8^ Evaluations and a recent career progress survey have highlighted the success of the MSc programmes, with, for example, at least 50% of recipients continuing with related work employed by research entities. Shifting to engagement with competitively selected LMIC institutions has further enabled achievements such as the creation of an active academic network leading to core curricula development of implementation research studies, with the aim to formalise the implementation research-profiled MSc degree in LMIC.^9^

## Democratising research, beyond academia and globally

Equitable participation in research to resolve bottlenecks and challenges in implementation is crucial for accurately addressing questions and to ensure representative research findings.^7^ Consequently, leadership, participation and benefits pertaining to research must include rich and meaningful representativeness from the health system; frontline practitioners, managers and policy makers, as well as civil society organisations, communities and particularly those known to be excluded from shaping research agendas or benefiting from their outcomes. Specifically, by building research capacity and leadership, countries can better understand structural or sociocultural barriers and find strategies to reduce them, leading to improved health equity. For instance, the Dodowa Health Research Centre in Ghana has coproduced evidence used in national policy for decades. These include disentangling community attitudes and early feasibility studies of the deployment of rectal artesunate against acute malaria in children under 5 years old. The research was integrated with capacity strengthening with broad involvement of different stakeholders and was successfully translated into policy making and established a national policy to impact the treatment of severe malaria in young children.^10^

It has been shown how sociocultural beliefs, structural norms and distrust in health systems or science can hamper vaccine delivery and uptake, for example, against yellow fever^11^ and how across vaccines and context, individuals’ trust in the government and society was key predictors of vaccine hesitancy.^12^ Research to resolve such bottlenecks must integrate stakeholders outside of academia to contribute to applicable solutions.

Equitable research involvement is vital to the global research ecosystem^13^ and research capacity strengthening can overcome inequities in health research participation and in health outcomes, worldwide.^14^ Conceptualising equitable research capacity and democratising research requires multipronged efforts within the global research community. Efforts should go beyond academia to ensure meaningful involvement and representativeness to ensure that a diverse and equitable group of scientists are involved in global agenda setting and researching critical innovations and developments and for the benefit of implementation-oriented research.

Democratisation of research, including researchers, communities and research participants, institutions and governance is critical to TDR’s strategy.^15^ TDR partners with seven competitively selected research training centres (RTC) in the regions, representing geographical and expertise diversity.^6^ The collaborations focus on the provision of individual and institutional capacity strengthening in implementation research, providing in-person, hybrid and online training across sub/regions, covering width and depth, along the end-to-end research spectrum. The network includes career mentoring and supervision of learning activities, projects and evidence-to-policy initiatives. A specific aim is to provide inclusive capacity strengthening among different professional groups and communities such as health professionals, policy makers and community representatives or health workers, in addition to researchers.^7^ These centres also contribute to the development and dissemination of TDR’s training resources.^16^,^18^

## Online access to research training

A key aspect of democratising research is providing access to research training beyond academia, with succinct, affordable and accessible modalities for learning. Courses need to remain flexible, rigorous and relevant.

To ensure wide dissemination of training opportunities, TDR and its regional RTC^6^ cocreate and organise no-cost, accessible, online courses to advance knowledge in implementation research applied to infectious diseases of poverty.^18^ In 2025, more than 14 000 participants registered for a variety of these implementation research and infectious disease-focused courses, in several languages, and with between 30% and 60% of learners completing one or more modules.

Challenges experienced among learners, in addition to using technology, included sustaining motivation and a lack of perceived context for future skills applications at individual and community levels.^19 20^ Learners want multilingual courses, with context and examples specific to a region or subregion. TDR trainings combine the ‘one size fits all’ of accessible online education with a diversified approach. Courses available in different languages, adding live online interactivity, and for select cohorts of learners, a combination with research integration and in-person training, is part of TDR and strategic capacity strengthening, reaching wider than training only. The democratisation of research also relies on efforts to ensure an equitable and diverse recruitment for research careers, building on equal access to in-depth learning, mentoring and leadership. The courses can serve as a gateway to engaging with research and to further professional development, where TDR efforts have been shown to be catalytic.^21^

## Case study: tuberculosis control in Indonesia

In 2010, the faculty of medicine, public health and nursing at Universitas Gadjah Mada became the WHO/TDR regional training centre for health research covering a South East Asian subregion. It has systematically formalised the use of implementation research to tackle pressing public health challenges. The approach resulted in a codeveloped national agenda with multiple stakeholders, without mandates driven by donors. A key outcome was a national strategy for implementation, with operational research and interdisciplinary teams designing context-specific research tied to national health priorities. These teams became instrumental when the Ministry of Health called for implementation research projects in 2023.

Beginning in 2017, the university partnered with the Mimika District TB Program, Timika Research Facility and Australia’s Menzies School of Health Research through the initiatives stratified approach to managing tuberculosis (TB) and planning, resource allocation, implementation, monitoring and evaluation of TB programmes.^22^ These projects scaled up household contact screening and preventive treatment for children under 5 years old, while strengthening programme information systems and applying a continuous quality improvement framework to close gaps in clinical and public health management. Efforts funded by the US Agency for International Development further supported active case finding and district action planning. The university used its implementation research capacity to design a theory-informed, mixed-methods protocol using frameworks such as the consolidated framework for implementation research, capability, opportunity, motivation and behaviour and reach, effectiveness, adoption, implementation and maintenance. From 2017, the team delivered group training, educational materials, mentoring and regular quality improvement meetings, where TB staff identified barriers and solutions.^23^

Between 2015 and 2019 in Mimika, annual TB case notifications rose 67% (1078–1796), with an 89% increase in bacteriologically confirmed cases (361–682). Primary care contributions to detection grew from 26% to 46% (277–822). Despite COVID-19 disruptions, case detection rebounded in 2021 and comorbidity screening and treatment adherence remained stable—showing the resilience of these system improvements driven by implementation research. These results show how sustained investment in implementation research can help to bridge the policy–practice gap and drive measurable gains in high-burden, resource-limited settings.

## Strengthening institutions and equitable partnerships

Confronting the infectious disease burden with existing and new interventions requires effective institutionalisation of research, applying a ‘one health’, multisectoral approach, including within health systems. Participation should include academia as well as health professionals and communities to identify and answer research questions.^7^ Important questions linked to themes such as climate’s impact on infectious diseases and antimicrobial resistance involve research capacity gaps that require closure among several disciplines and functions. Strong academic research institutions and national and global entities engaged in governance, health programmes and evidence-based policy making are needed to resolve these challenges.

Categorisations of partners, and subsequent allocations of research capacity-strengthening efforts, have traditionally been made based on a dichotomous view of LMIC recipients versus HIC donors. This perspective risks losing sight of the heterogeneous spectrum of actors within LMIC, which have hugely different research and capacity needs.

A focus on partnerships driven by academic excellence risks missing the individuals or institutions most in need. A general phenomenon in complex human networks formed between, for example, individual scientists or research partners, is what is known as ‘preferential attachment’. The phenomenon is often described as ‘the rich get richer’, implying that a person with existing wealth is also more likely to attract more of the same. Similarly, an institution or individual researcher with more partnerships has a higher probability of attracting new ones.^24^ The risk of losing an inclusive and wide focus in favour of the exclusive partnerships needs to be recognised and addressed.

Individual researchers may also be disadvantaged by inequities and intersectional gender effects. Traditional gender roles and professional power inequalities are often preserved by academic hierarchies.^25 26^ Fee-based university tuition and gate-keeping entry systems such as requirements of recommendations from recognised institutions or individuals mostly discriminate against lower socioeconomic groups and are toughest to overcome for women. Inequities at undergraduate university levels keep increasing up to academic, research and healthcare leadership.^25^,^27^

Similarly, institutions located in resource poor or rural areas with challenged educational systems, and where national governance and supportive research policy are weak, face inequities regarding institutional scientific achievements that may further diminish possibilities of competitive grants or recruitment of the best researchers or partners.

Diversifying power and drawing from the strengths of flagship institutions, to support equity and the most in-need institutions or individuals, is a critical aim of TDR capacity strengthening. It requires having contextualised knowledge rather than making generalised assumptions, applying, for example, preferential selections, or selective targeting related to gender, resources or geographical context, institutional financing, an individual’s undergraduate or postgraduate affiliation and more. A critical take on the risk of homogeneity of existing partnerships can illuminate how to leverage long-term partnerships, while disseminating the capacity-building aim and mitigating intersectional inequities.

TDR has often worked in established partnerships, for example, with its regional research training centres.^6^ A critical aim has been the wide dissemination of research capacity-building through partner networks. TDR has selected these regional research training centres in part for their potential to engage and support institutions in heterogeneous regional or geographic contexts. Accessible research capacity-strengthening initiatives outside established partnerships have been a goal, and collaborations aim to include less established institutions outside urban areas, with institutional and individual mentoring to promote equity.

Another priority has been gender-responsive options, critical to women facing demands based on traditional gender roles that limit career development. These include, for example, provision of online courses that allow flexibility in time and place of training and ‘sandwich models’,^28^ where capacity building, training and research abroad are combined with activities linked to the home institution, as in TDR’s online and hybrid course programme,^67 16^,^18^ and its clinical research leadership programme.^29^

## Case study: training in Latin America

The regional research training centre for the Latin America and Caribbean region, based at Centro Internacional de Entrenamiento e Investigaciones Medicas in Colombia, has been supported by TDR since 2009 to run a capacity-building programme.^16^ The programme trains researchers and practitioners in a range of skills required to conduct research, with a particular emphasis on good health research practices (GHRP), effective planning, monitoring and evaluation (EPPE) and implementation research. Participants develop their own research proposals with guidance from experienced mentors and are encouraged to become trainers themselves—building institutional capacity and fostering peer learning.

By March 2025, the TDR Latin American Regional Training Center had delivered over 110 courses, trained over 1500 professionals and certified over 100 trainers to lead training in their own institutions. A successful example is in Honduras. Over the years, more than 60 professionals from various institutions, including the National Autonomous University of Honduras (NAUH), the Medical College of Honduras and the Antonio Vidal Institute of Infectious Diseases and Parasitology, have been trained in GHRP, EPPE and implementation research. About 15 became trainers, and at least four of them have replicated or adapted the training in their own institutions or with other groups, showing the model’s scalability and adaptability. The model has also supported capacity building in higher education; the NAUH, for example, has integrated an EPPE module into its masters in public health programme.

Despite progress, the sustainability of the strategy continues to face challenges. Long-term institutionalisation requires strong commitment of universities and health programmes as well as the recognition of trained facilitators as ‘champions’ within their institutions and discipline, where education needs be a recognised part of career development. Identifying and retaining key individuals is essential. Moreover, maintaining an active regional training network depends on trainers and participating institutions having sufficient resources, dedicated time and adequate working conditions, particularly in contexts where professionals balance multiple responsibilities.

Key factors that have contributed to the TDR Latin American Regional Training Center’s strategy’s success include its alignment with national and regional health priorities, close collaboration with partner institutions such as national health authorities and universities. The sustained support from international organisations such as TDR and the Pan American Health Organization and the use of virtual and blended training formats to reduce costs and promote more equitable participation.

## Challenges and opportunities

Remaining challenges include reaching and engaging underserved research communities, specifically in lower resourced countries facing the highest burden and most severe consequences of endemic and epidemic infectious diseases. Language barriers, for example, in francophone Africa or Latin America, lack of national support to research and limited resources for academia and limited national and international academic network engagement make the case for sustained support to research capacity strengthening. The risk of fuelling unequal power hierarchies can be counteracted with strategic interventions that trigger subsequent actions, collaborations and knowledge-sharing processes beyond the initial scope, thereby amplifying and sustaining their benefits over time, as illustrated in the ripple effects of the train-the-trainers model above. Navigating unfulfilled needs of research capacity and vast resource gaps with equitable partnerships paired with explicit dissemination strategies can continue to make a difference.

**Recommendations**

1. Shift research capacity strengthening from externally driven models to approaches that support national and regional leadership in setting research agendas, designing studies and applying findings to address context-specific health challenges.
2. Build long-term, equitable collaborations that include not only academic institutions of excellence but also leverage these to include also underserved and least-resourced institutions. These partnerships should be designed to bridge gaps between research and practice and to allow access to research tools among diverse health professionals and communities.
3. Develop and support flexible training models, mentorship and targeted efforts for underrepresented groups to tackle intersectional gender inequities.
4. Democratise research capacity by providing accessible, multilingual and flexible training and skills development opportunities for a broad range of stakeholders, including health professionals, policy makers and community members.
5. Institutionalise capacity strengthening for sustainability to enhance diversity in science across local and global research agendas: encourage the development of inclusive networks and mentoring systems to ensure that gains in capacity are maintained and scaled over time, ensuring that diverse voices shape research priorities and benefit from research outcomes.

## Conclusions

This article emphasises the evolution from externally driven models centred on HIC towards inclusive, context-sensitive approaches that prioritise local ownership, diversity and sustainability. The paradigm shift from ‘imposing technical support’ to ‘fostering ownership of knowledge’ has catalysed new models of engagement, such as strengthening implementation research capacity among health professionals and communities beyond academia, and regionally based postgraduate training focused on core competencies in implementation research.

Institutionalised, inclusive research can align with national priorities and yield measurable health outcomes. However, persistent inequities—rooted in gender, geography and institutional hierarchies—continue to constrain participation and impact. Addressing these requires deliberate strategies to democratise access, diversify partnerships and support under-represented institutions and individuals. The continuum between academic collaboration and capacity strengthening must be explicitly acknowledged, with dynamic, needs-based roles replacing static donor-recipient models. Moving forward, equitable research capacity strengthening must be embedded in governance structures, supported by sustainable resources, and guided by inclusive networks that reflect the complexity of global health needs. Only through such systemic and inclusive approaches can research serve as a driver of health equity and development.

## Data Availability

No data are available.
